# Intestinal carriage of multidrug-resistant bacteria among healthcare professionals in Germany

**DOI:** 10.3205/id000033

**Published:** 2017-11-22

**Authors:** Katalin Jozsa, Katja de With, Winfried Kern, Claudia Reinheimer, Volkhard A. J. Kempf, Cornelia Wichelhaus, Thomas A. Wichelhaus

**Affiliations:** 1 Institute of Medical Microbiology and Infection Control, Hospital of Goethe-University, Frankfurt am Main, Germany; 2 Universitätsklinikum Carl Gustav Carus, Zentralbereich Klinische Infektiologie, Dresden, Germany; 3 Division of Infectious Diseases, Department of Medicine, University Medical Center, Freiburg i.Br., Germany; 4 Department of Mathematics, Technical University of Darmstadt, Darmstadt, Germany

**Keywords:** healthcare professional, ESBL, MRSA, VRE, colonization

## Abstract

Healthcare professionals (HCP) might be at increased risk of acquisition of multidrug-resistant bacteria (MDRB), i.e., methillicin-resistant *Staphylococcus aureus* (MRSA), vancomycin-resistant enterococci (VRE), and multidrug-resistant gram-negative bacteria (MDRGN) and could be an unidentified source of MDRB transmission.

The aim of this study was to determine the prevalence as well as risk factors of MDRB colonization among HCP.

HCP (n=107) taking part in an antibiotic stewardship program, were voluntarily recruited to perform a rectal swab and to fill in a questionnaire to identify risk factors of MDRB carriage, i.e. being physician, gender, travel abroad within the previous 12 months, vegetarianism, regular consumption of raw meat, contact to domestic animals, household members with contact to livestock, work or fellowship abroad, as well as medical treatment abroad and antibiotic therapy within the previous 12 months.

Selective solid media were used to determine the colonization rate with MRSA, VRE and MDRGN. MDRGN were further characterized by molecular analysis of underlying β-lactamases.

None of the participants had an intestinal colonization with MRSA or VRE. 3.7% of the participants were colonized with extended-spectrum beta-lactamase (ESBL)-producing *Enterobacteriaceae*, predominantly *bla*_CTX-M_ type. Neither additional flouroquinolone resistance nor carbapenem resistance was detected in any of these isolates. No risk factors were identified to have a significant impact of MDRB carriage among HCP.

A colonization rate of 3.7% with ESBL-producing *Enterobacteriaceae* is of interest, but comparing it to previously published data with similar colonization rates in the healthy population in the same geographic area, it is probably less an occupational risk.

## Introduction

Infection and colonization with multidrug-resistant bacteria (MDRB) such as methillicin-resistant *Staphylococcus aureus* (MRSA), vancomycin-resistant enterococci (VRE), and multidrug-resistant gram-negative bacteria (MDRGN) are associated with increased mortality, morbidity and hospital costs [1], [2], [3].

*Staphylococcus aureus *is part of the normal human flora. A study reports that about 20% of high risk patients, i.e. patients from nursing homes, have an intestinal colonization with *S. aureus* and about 9% of the patients are colonized with MRSA [4]. Nasal carriage seems to be a predisposition for intestinal carriage [4]. MRSA carrier can be categorized into transient and persistent carriers. Persistent carriers are described to be colonized on several sites over months or years. They are more likely to have endogenous infections due to their MRSA strain compared to transient carriers [5], [6]. Persistent carriers are defined by remaining colonized unless they are treated with an effective agent, in comparison to transient carriers, who are described to clean their colonization even in the absence of an effective treatment [5]. Transmission frequently results from the transient colonization through the hands of hospital staff, carrying strains from one to the other patient [7]. 

VRE is the third most common nosocomial agent in the German healthcare setting, showing an increased prevalence from 9.3% in 2008 to 18.5% in 2012 in hospital settings [8]. The number of nosocomial infections with VRE increased from 3.9% in 2007/2008 to 7.2% in 2013/2014 [9]. In the neighbouring countries of Germany, the prevalence of VRE has been described to be lower, e.g. Denmark 2.0%, the Netherlands 0.0%, France 0.8%, Belgium 1.4%, Austria 3.2% [8].

MDRGN and in particular ESBL-producing *Enterobacteriaceae* are reported to colonize patients at risk as well as healthy people with increasing frequency [10]. The introduction of third generation cephalosporins in the 1980s [11] was a milestone in the treatment of gram-negative infections. This increase of selective pressure might also have promoted the spread of resistant organisms throughout Europe. The first cases of extended-spectrum β-lactamase producing* Klebsiella pneumoniae* and *Serratia marcescens* isolates were published in Germany in 1983 [12], [13]. According to EARS-data, the percentage of invasive* E. coli* isolates resistant to third generation cephalosporins increased from 0% in year 2000 to 10.4% in year 2015 in Germany [14]. 

A study conducted on the prevalence of fecal carriage in the healthy population in Germany between October 2009 and November 2012, demonstrated a frequency of 6.3% ESBL-producing *E. coli*, predominantly (95,1%) *bla*_CTX-M_ type [15].

The aim of this study was to investigate the prevalence of intestinal MDRB colonization in healthcare professionals (HCP) and to identify potential risk factors of MDRB carriage.

## Materials and methods

HCP, who took part in the antibiotic stewardship program supported by the German Society for Infectious Diseases, the divisions of Infectious Diseases Freiburg and Dresden, were recruited from March 2013 till March 2014. Participation was voluntary and anonymous. Volunteers were asked to perform a rectal swab and to fill out a questionnaire. This questionnaire addressed the potential risk factors of MDRB carriage, i.e. being physician, gender, travel abroad, vegetarianism, consumption of raw meat, contact to domestic animals, household members with contact to livestock, work or fellowship abroad, as well as medical treatment abroad and antibiotic therapy within the previous 12 months.

### Detection of MDRB

The rectal swab was inoculated into 500 µl 0.9% NaCl suspension. Each culture media was inoculated with 20 µl suspension. Media used were Endo-Agar with Cefuroxim-disk, selective CHROMagar™ ESBL plates (Mast Diagnostica, Paris, France), chromID CARBA (bioMérieux, Nürtingen, Germany), chromID OXA-48 (bioMérieux), chromID VRE (bioMérieux), Brilliance MRSA-Agar (Oxoid, Wesel, Germany). When growth was detected, species identification was performed by matrix-assisted laser desorption ionization time-of-flight (MALDI-TOF) or biochemical reactions performed by VITEK2 (bioMérieux), additionally antibiotic susceptibility testing was performed according to Clinical and Laboratory Standards Institute (CLSI) guidelines M100-S24 (Version January 2014) by VITEK2 (bioMérieux), agar diffusion (Oxoid) or antibiotic gradient tests (Liofilchem, Roseto degli Abruzzi, Italy).

### Characterization of MDRGN

MDRGN is defined as *Enterobacteriaceae* with extended spectrum beta-lactamase (ESBL)-phenotype as well as *Enterobacteriaceae*, *Pseudomonas aeruginosa*, and *Acinetobacter baumannii* resistant against at least piperacillin, any 3^rd^/4^th^ generation cephalosporin and ciprofloxacin [16], [17]. ESBL phenotype was identified by VITEK 2 Advanced Expert System™ (bioMérieux). ESBL encoding *bla* genes, i.e. *bla*_CTX-M_, *bla*_TEM_ and *bla*_SHV_, were detected via PCR and subtype analysis was done by subsequent sequencing, as published by Dallenne C. et al. [18]. 

### Statistical analysis

Categorical data i.e. being physician, gender, travel abroad, vegetarianism, consumption of raw meat, contact to livestock and contact to domestic animals, work or fellowship abroad, as well as medical treatment abroad and antibiotic therapy within the previous 12 months, were analyzed using Fisher’s exact test with the exception for datasets where a cell equaled zero. In these cases, the Pearson, Mantel and Haenszel chi-squared test was applied using a continuity correction. Odds ratios (OR) and 95% confidence intervals (CI) are given. All tests were performed two-tailed, and a *p*-value ≤0.05 was considered as statistically significant. 

### Ethics

After consultation with the head of the ethic committee at the Goethe University Hospital Frankfurt no further approval for this survey was deemed necessary.

## Results

### Prevalence and characterization of MDRB

Between March 2013 and March 2014, 107 HCP were screened for intestinal carriage of MDRB. 69.1% (n=74) and 30.7% (n=33) of participants were physicians and non-physician healthcare professionals, respectively. Neither MRSA nor VRE colonization was detected in HCP. 

3.7% (n=4) (confidence interval 95% (CI) =1.02–9.29) of participants were tested positive for ESBL-producing *E. coli*. ESBL enzyme types were determined to be *bla*_CTX-M-1_ (n=2) and *bla*_CTX-M-14_ (n=2). Neither additional flouroquinolone resistance nor carbapenem resistance was detected in any of these isolates. 

Neither multidrug-resistant* A. baumannii* nor *P. aeruginosa* were detected among HCP. 

### Risk factors associated with MDRB

Statistical analysis to identify risk factors associated with MDRB colonization is summarized in Table 1 (Tab. 1). No risk factor for colonization with ESBL producing *Enterobacteriaceae* could be identified.

## Discussion

Antibiotic resistance is recognized as a major threat to modern medicine [19]. Patients infected by a MDRB have a higher mortality and an increased morbidity, leading to a boost of costs [1], [2], [3]. Since intestinal colonization with MDRB is one of the most frequent reservoirs of infections [20], we intended to investigate the prevalence of intestinal colonization with MDRB in HCP. Fecal carriage of MDRB in the population has previously been described in several studies, e.g. by Valenza et al. in 2014 [15] reporting 6.3% of ESBL-producing *Enterobacteriaceae* colonization in Germany. Furthermore Meyer et al. found a rate of 3.5% ESBL-producing *Enterobacteriaceae* carriage among healthy infection control personnel in 2011 [21]. In our setting, we found a prevalence of 3.7% ESBL carriage among HCP, which is consistent with the published data [15], [21]. 

Molecular characterization of ESBL-encoding genes revealed the presence of *bla*_CTX-M-1_ and *bla*_CTX-M-14_. This result reflects the published predominance of these genes together with *bla*_CTX-M-15_ in Germany [22] and also is in accordance with epidemiological studies that reported the shift from TEM and SHV enzymes being highly prevalent until the 1990s to CTX-M group enzymes in Europe [15], [22], [23], [24], [25]. 

The prevalence of ESBL-producing *Enterobacteriaceae* geographically varies. Geser et al. found that 5.8% staff members of a meat-processing company in Switzerland were colonized with ESBL [24]. A Swedish study showed a prevalence of ESBL-producers of 2.9% among healthy preschool children in 2010 [26]. Data from France showed a prevalence of ESBL-producing *Enterobacteriaceae* carriers in 2009 and 2011 of 2.1% and 6%, respectively [27], [28]. The prevalence of ESBL-producing *Enterobacteriaceae* carriage is much higher in Asia than Europe. Li et al. found a prevalence of ESBL-producing *E. coli* of 50.5% in healthy individuals in China in 2011 [25], predominantly harbouring CTX-M enzymes. Another study from Thailand observed a prevalence of 61.7% *Enterobacteriaceae* showing an ESBL phenotype and 58.2% out of total 160 participants had a CTX-M enzyme [29].

Several studies [30], [31] investigated the acquisition of ESBL-producing *Enterobacteriaceae* among participants during travel. Post-travel analysis demonstrated that 35% of the participants were colonized with a new ESBL strain during travel, mostly acquired in South-East Asia region [30].

In this study, no significant risk factor associated with the carriage of ESBL-producing *Enterobacteriaceae* was identified. Though some participants matched risk factors which have previously been described in the literature, i.e. travel [30], [31], companion animals or livestock contact [21]. In our study, neither travel history nor work or fellowship abroad in the previous 12 months could be described as a significant risk factor for ESBL carriage. 

Limitation of our data is that we investigated only a small number of participants in a cohort of HCP who might be sensitized to hand hygiene and infection control measurements, and might therefore not be representative for all HCP.

## Conclusion

In summary, we indentified a colonization rate of 3.7% of ESBL-producing *Enterobacteriaceae* in HCP. Since this colonization rate does not differ from the healthy population in this geographical area and no other MDRB have been identified in this cohort, HCP presumably do not have an occupational risk of MDRB colonization. 

## Notes

### Competing interests

The authors declare that they have no competing interests.

### Acknowledgements

We thank Denia Frank for excellent technical support.

## Figures and Tables

**Table 1:**
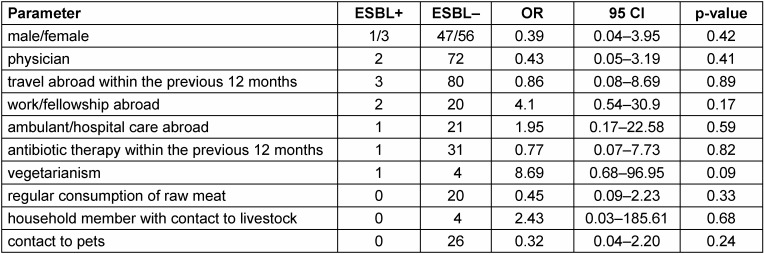
Risk factors associated with ESBL-producing *Enterobacteriaceae*
